# Feasibility of At-Home Serial Testing Using Over-the-Counter SARS-CoV-2 Tests With a Digital Smartphone App for Assistance: Longitudinal Cohort Study

**DOI:** 10.2196/35426

**Published:** 2022-10-18

**Authors:** Carly Herbert, John Broach, William Heetderks, Felicia Qashu, Laura Gibson, Caitlin Pretz, Kelsey Woods, Vik Kheterpal, Thejas Suvarna, Christopher Nowak, Peter Lazar, Didem Ayturk, Bruce Barton, Chad Achenbach, Robert Murphy, David McManus, Apurv Soni

**Affiliations:** 1 Program in Digital Medicine Department of Medicine University of Massachusetts Chan Medical School Worcester, MA United States; 2 Department of Emergency Medicine University of Massachusetts Chan Medical School Worcester, MA United States; 3 UMass Memorial Medical Center Worcester, MA United States; 4 National Institute of Biomedical Imaging and Bioengineering Bethesda, MD United States; 5 National Institutes of Health Bethesda, MD United States; 6 Division of Infectious Disease and Immunology Departments of Medicine and Pediatrics University of Massachusetts Chan Medical School Worcester, MA United States; 7 CareEvolution, LLC Ann Arbor, MI United States; 8 Department of Population and Quantitative Health Sciences University of Massachusetts Chan Medical School Worcester, MA United States; 9 Division of Infectious Disease Department of Medicine Feinberg School of Medicine, Northwestern University Chicago, IL United States; 10 Department of Cardiology University of Massachusetts Chan Medical School Worcester, MA United States

**Keywords:** COVID-19, SARS-CoV-2, rapid tests, MyDataHelps smartphone app, mHealth, mobile health, serial self-testing, digital health, pandemic, self test

## Abstract

**Background:**

The ongoing SARS-CoV-2 pandemic necessitates the development of accurate, rapid, and affordable diagnostics to help curb disease transmission, morbidity, and mortality. Rapid antigen tests are important tools for scaling up testing for SARS-CoV-2; however, little is known about individuals’ use of rapid antigen tests at home and how to facilitate the user experience.

**Objective:**

This study aimed to describe the feasibility and acceptability of serial self-testing with rapid antigen tests for SARS-CoV-2, including need for assistance and the reliability of self-interpretation.

**Methods:**

A total of 206 adults in the United States with smartphones were enrolled in this single-arm feasibility study in February and March 2021. All participants were asked to self-test for COVID-19 at home using rapid antigen tests daily for 14 days and use a smartphone app for testing assistance and to report their results. The main outcomes were adherence to the testing schedule, the acceptability of testing and smartphone app experiences, and the reliability of participants versus study team’s interpretation of test results. Descriptive statistics were used to report the acceptability, adherence, overall rating, and experience of using the at-home test and MyDataHelps app. The usability, acceptability, adherence, and quality of at-home testing were analyzed across different sociodemographic, age, and educational attainment groups.

**Results:**

Of the 206 enrolled participants, 189 (91.7%) and 159 (77.2%) completed testing and follow-up surveys, respectively. In total, 51.3% (97/189) of study participants were women, the average age was 40.7 years, 34.4% (65/189) were non-White, and 82% (155/189) had a bachelor’s degree or higher. Most (n=133/206, 64.6%) participants showed high testing adherence, meaning they completed over 75% of the assigned tests. Participants’ interpretations of test results demonstrated high agreement (2106/2130, 98.9%) with the study verified results, with a κ score of 0.29 (*P*<.001). Participants reported high satisfaction with self-testing and the smartphone app, with 98.7% (157/159) reporting that they would recommend the self-test and smartphone app to others. These results were consistent across age, race/ethnicity, and gender.

**Conclusions:**

Participants’ high adherence to the recommended testing schedule, significant reliability between participants and study staff’s test interpretation, and the acceptability of the smartphone app and self-test indicate that self-tests for SARS-CoV-2 with a smartphone app for assistance and reporting is a highly feasible testing modality among a diverse population of adults in the United States.

## Introduction

Since the emergence of the SARS-CoV-2 pandemic in late 2019, more than 590 million cases and 6.5 million deaths from COVID-19 have been reported worldwide [1]. Over 2 years into the pandemic, the United States continues to face waves of increasing SARS-CoV-2 cases. The ongoing pandemic necessitates the development of accurate, rapid, and affordable diagnostics to help curb SARS-CoV-2 disease transmission, morbidity, and mortality, as well as safely navigate social re-engagement [2].

Antigen-based rapid diagnostic tests (Ag-RDTs) detect SARS-CoV-2 viral proteins in multiple specimen types and facilitate opportunities for large-scale, cost-effective testing solutions [3,4]. Ag-RDTs are preferred by over two-thirds of SARS-CoV-2 test users, especially in comparison to molecular tests, which can take days to receive the result [5,6]. Numerous Ag-RDTs for SARS-CoV-2 have received Emergency Use Authorization by the US Food and Drug Administration for point-of-care testing in the health care setting and, more recently, for at-home use, with evidence consistently showing the validity of self-collected specimens for SARS-CoV-2 testing [7,8]. Self-testing at home offers great opportunity for scaling up and implementing regular testing of both asymptomatic and symptomatic individuals, a key step toward controlling the COVID-19 pandemic [2,9-11]. Furthermore, self-testing offers the opportunity to increase testing access across geographic, sociodemographic, and socioeconomic groups to improve health outcomes and reduce health care disparities [12-14]. However, little is known about individuals’ use of rapid antigen tests at home and how to facilitate the user experience [5,15].

The objectives of this study were to describe the feasibility of the at-home use of rapid antigen tests for SARS-CoV-2, as well as participants’ use of the MyDataHelps smartphone app (CareEvolution) to support at-home testing. This study aimed to describe the usability and acceptability of self-tests for SARS-CoV-2, variation in use across different sociodemographic and socioeconomic groups, and how participants interact with the MyDataHelps smartphone app to report symptoms and test results. We hypothesized that the acceptability and usability of the rapid antigen tests and smartphone app would be consistent across sociodemographic and socioeconomic groups.

## Methods

### Study Population

Participants were recruited from the University of Massachusetts (UMass) Chan Medical School and Northwestern University using best practices developed by the RADx Tech Community Health Equity and Engagement Team to maximize the representation of diverse age, sex, race, ethnicity, education, and socioeconomic groups [16]. Participants were enrolled in the study during February and March 2021. For inclusion in the study, individuals were required to be aged ≥18 years, be willing to use their own smartphone device and download the MyDataHelps app, have reported no symptoms attributable to COVID-19 within 48 hours prior to screening, and be proficient in English.

### Ethics Approval

Details of the study procedures were explained to participants, and written informed consent was obtained from all participants. This study was approved by the Institutional Review Board (H00022342) at UMass Chan Medical School and Northwestern University, which had a reliance agreement with the UMass Chan Medical School Institutional Review Board.

### Study Procedures

All participants were asked to self-test for COVID-19 at home daily over a consecutive 14-day period. Participants were mailed a QuickVue test kit (Quidel) containing testing supplies for 25 tests; written testing instructions; and a prepaid, pre-addressed return box for test strips with return instructions. All test kits used anterior nasal swabs, and instructions directed participants on how to properly swab their nasal cavity. Participants were given access to the MyDataHelps app to support testing. The MyDataHelps app allowed participants to view testing instructions, report test results, verify test results with the study team, track their testing history, respond to surveys, and access the study team’s contact information (Multimedia Appendix 1).

Participants were informed that they could report their test results to the study team either digitally through the MyDataHelps app or through manual written recording. If participants opted to use the MyDataHelps app for reporting test results, they were asked to report their interpretation of the test results—positive or negative—and upload an image of the test strip (Multimedia Appendix 1). Study coordinators validated all test results using the test strip image, with digital verification occurring within 24 hours of reporting. Written test results were mailed to study coordinators along with all test strips for verification at the end of the study. Participants were instructed to contact study coordinators with any questions during the study period. All interactions between study coordinators and participants were recorded in a contact log.

If a participant showed COVID-19 symptoms, reported close exposure to a person positive for SARS-CoV-2, or tested positive on a home test, the study team contacted the participant and scheduled confirmatory SARS-CoV-2 polymerase chain reaction testing by trained personnel using established protocols and procedures. If an individual tested positive for SARS-CoV-2 on confirmatory testing, participants were removed from the study and received an exit survey on the day of the positive results out of safety for the participants.

### Study Questionnaires

Questionnaires were administered to participants through the MyDataHelps app. Eligible and consenting study participants were given a baseline survey, daily surveys, a midpoint survey, and an exit survey. The baseline survey assessed their sociodemographic characteristics, anthropometrics, health status, and social engagement. Health status included questions regarding disability, pregnancy, current alcohol and cigarette use, chronic conditions, and report of common COVID-19 symptoms. Daily surveys asked for self-interpretation of the test results (positive or negative), image upload of the test strip, and symptom reporting. The midpoint survey was given to participants on day 7, and participants were asked to self-report the total number of completed tests and their social engagement practices. Lastly, the exit survey, on day 14 of the study, asked for a self-report of the total number of tests completed, acceptability and experience of using the MyDataHelps app, acceptability of the at-home test, social engagement, insurance status (no insurance, private, or public insurance), and health status. The acceptability of the at-home test was assessed by asking participants if they would recommend the self-test to someone else using the Net Promotor Scale. The number of tests reported and number of daily image uploads over the 14-day testing period determined adherence to the testing schedule. Adherence to the testing schedule was classified into 4 categories: no (0%) adherence, low (<50%) adherence, moderate (50% to 75%) adherence, and high (>75%) adherence. Using a Likert scale, the experience and acceptability of the MyDataHelps app was assessed by asking for the participants’ overall rating of the app (1=one of the worst apps I’ve used to 5=one of the best apps I’ve used). Participants were asked how often they had difficulties using the app (1=all the time to 5=never), the usefulness of different features of the app (1=really useful to 5=really useless), whether they would recommend the app to another person to help them perform an at-home test for COVID-19 (1=definitely yes to5=definitely not), and if they would continue using the app to keep testing themselves at home for COVID-19 (1=definitely yes to 5=definitely not).

### Data Analysis

Descriptive statistics were used to report the acceptability, adherence, overall rating, and experience of using the at-home test and MyDataHelps app. The usability, acceptability, adherence, and quality of at-home testing were analyzed across different sociodemographic, age, and educational attainment groups to evaluate the impact on existing socioeconomic disparities, using ANOVA to evaluate significance. The study coordinator contact log was text mined and used to generate a word cloud in R statistical software (version 4.2.1; R Foundation for Statistical Computing) to characterize participant interactions with study staff.

## Results

### Participant Characteristics

A total of 206 participants enrolled in the study during February and March 2021. There were 5 (5/206, 2.4%) participants who tested positive for SARS-CoV-2 during the study period, and they were removed from the study. Of the 206 participants, 189 (91.7%) and 159 (77.2%) completed testing and follow-up surveys, respectively. Among participants who completed testing, slightly more than half (97/189, 51.3%) were women, the average age of the study population was 40.7 years, 34.4% (65/189) were non-White, and 82% (155/189) had a bachelor’s degree or higher (Table 1). At the time of study enrollment (February 2021), only 2.5% of the US population were fully vaccinated for SARS-CoV-2 [17].

**Table 1:** Participant characteristics stratified by testing adherence.

Characteristic		Adherence to daily testing		
		Low (n=19), n (%)	Moderate (n=37), n (%)	High (n=133), n (%)
**Age (years)**				
	18-39	11 (57.9)	24 (64.9)	62 (46.6)
	40-64	3 (15.8)	13 (35.1)	49 (36.8)
	≥65	2 (10.5)	0 (0)	18 (13.5)
	No answer	3 (15.8)	0 (0)	4 (3)
**Gender**				
	Male	8 (42.1)	12 (32.4)	62 (46.6)
	Female	8 (42.1)	24 (64.9)	65 (48.9)
	Nonbinary or transgender	1 (5.3)	1 (2.7)	3 (2.3)
	No answer	2 (10.5)	0 (0)	3 (2.3)
**Race/ethnicity**				
	Hispanic	2 (10.5)	8 (21.6)	12 (9)
	Non-Hispanic Asian	1 (5.3)	5 (13.5)	13 (9.8)
	Non-Hispanic Black	2 (10.5)	4 (10.8)	15 (11.3)
	Non-Hispanic Other	1 (5.3)	0 (0)	2 (1.5)
	Non-Hispanic White	11 (57.9)	20 (54.1)	89 (66.9)
	No answer	2 (10.5)	0 (0)	2 (1.5)
**Education level**				
	Master’s degree or higher	7 (36.8)	7 (18.9)	34 (25.6)
	Bachelor’s degree or equivalent	6 (31.6)	24 (64.9)	77 (57.9)
	High school or lower	4 (21.1)	5 (13.5)	19 (14.3)
	No answer	2 (10.5)	1 (2.7)	3 (2.3)
**Employment status**				
	Working now	10 (52.6)	28 (75.7)	94 (70.7)
	Student	2 (10.5)	5 (13.5)	10 (7.5)
	Retired	3 (15.8)	1 (2.7)	19 (14.3)
	Other	4 (21.1)	3 (8.1)	10 (7.5)

### Patient-Reported Usability and Acceptability of At-Home Testing

**Figure 1:**
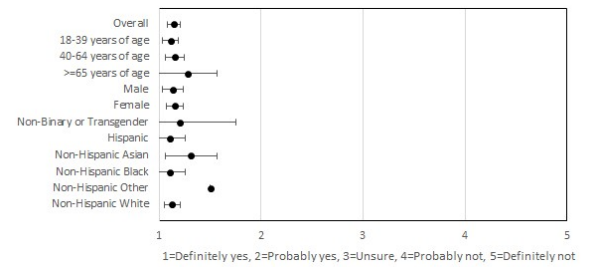
Usability and acceptability of self-tests.

In all, 91.7% (189/206) of the participants performed 1 or more tests during the study period (Table 2). The majority (133/206, 64.6%) of the participants showed high adherence to testing and picture upload, characterized as testing and uploading the picture of the test strip to the app on more than 75% of the indicated days (Table 1). Participants aged 18-39 years comprised the majority of the moderate (24/37, 64.9%) and low (11/19, 57.9%) adherence groups, whereas 90% (18/20) of the participants aged ≥65 years reported high adherence (*P*=.03; Table 1). Comparatively, only 63.9% (62/97) and 75.4% (49/65) of participants aged 18-39 years and 40-64 years demonstrated high adherence, respectively. Participants’ interpretations of test results demonstrated high agreement (2106/2130, 98.9%) with the study verified results, with a κ score of 0.29 (*P*<.001; Table 3). Overall, participants reported high satisfaction with at-home testing, with 98.7% (157/159) of the participants reporting that they would definitely or likely recommend the self-test to others (Figure 1).

**Table 2:** Total number of tests performed in the 14-day study period.

Total number tests performed in the 14-day study period	Participants (N=206), n (%)
0	17 (8.3)
1	2 (1)
2	3 (1.5)
3	2 (1)
4	2 (1)
5	2 (1)
6	2 (1)
7	6 (2.9)
8	7 (3.4)
9	9 (4.4)
10	22 (10.7)
11	24 (11.7)
12	32 (15.5)
13	35 (17)
14	41 (19.9)

**Table 3:** Reliability of self-interpretation versus study verification of at-home antigen-based rapid diagnostic tests.

Self-interpretation	Study verification			
	Negative	Positive	Invalid	Total
Negative	2102	5	11	2118
Positive	4	4	2	10
Invalid	2	0	0	2
Total	2108	9	13	2130

### MyDataHelps Participant Usability

**Figure 2:**
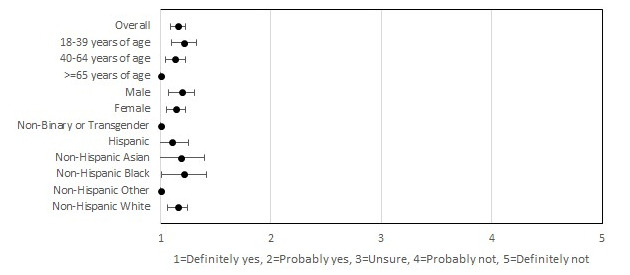
Participants’ willingness to recommend smartphone app to others.

**Figure 3:**
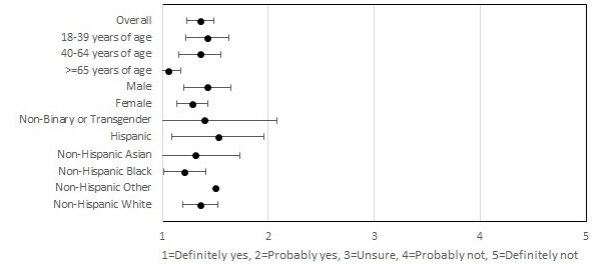
Participants’ interest in continuing to use the smartphone app after the study period.

**Figure 4:**
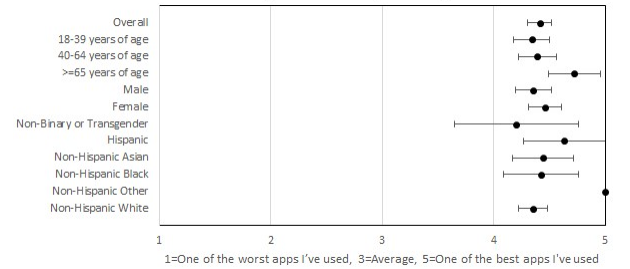
Participants’ overall rating of the smartphone app.

Participants also reported high satisfaction with the MyDataHelps app. In all, 98.7% (157/159) of the participants indicated that they would definitely or probably recommend the app to others, with 91.8% (146/159) indicating that they would continue using the app for at-home testing if possible (Figures 2 and 3). These results were consistent across all age, race/ethnicity, and gender groups. In all, 77.4% (123/159) of the participants reported never having difficulties using the app, and 3.8% (6/159) reported having difficulties with the app most or all of the time. Among participants who reported difficulties, internet connection issues (5/6, 83%) were the most common reason. Participants on average found the COVID-19 testing instructions to be the most useful feature of the app, with 88.1% (140/159) of the participants finding this feature “very useful.” The overall rating of the app was 4.4 out of 5, and the overall rating did not differ by age, gender, or race/ethnicity (Figure 4).

### Participant and Study Coordinator Interactions

**Figure 5:**
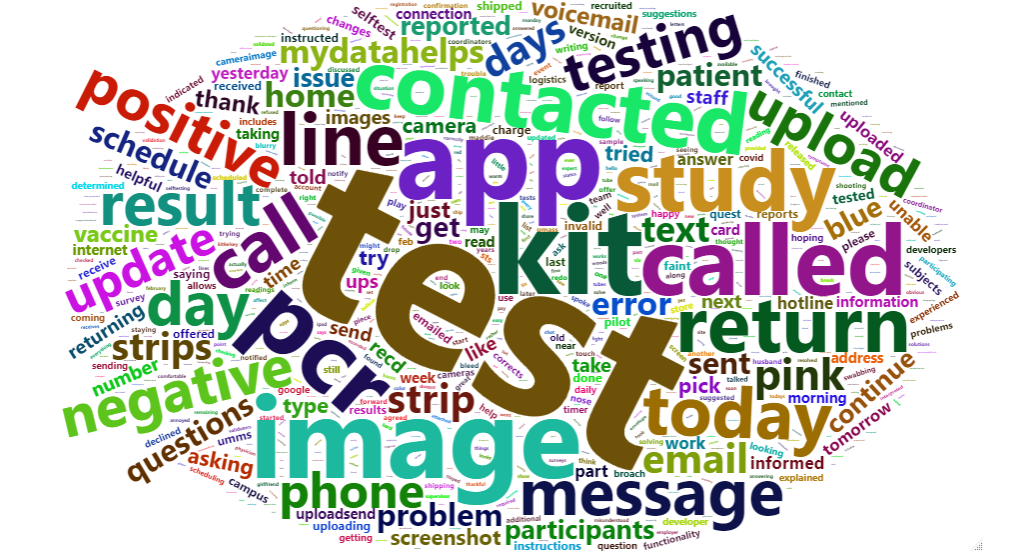
Word cloud of participant interactions with study coordinators.

Over the study period, there were a total of 117 phone and email interactions between study staff and participants. The most discussed topics were test kit return (43/117, 36.8%), test results (28/117, 23.9%), image upload (21/117, 17.9%), and scheduling confirmatory polymerase chain reaction testing (25/117, 21.4%; Figure 5). These topics were not mutually exclusive.

## Discussion

### Principal Findings

We described the feasibility of at-home rapid antigen testing for COVID-19 using a mobile app for testing support. Most participants displayed high adherence to the recommended testing schedule and were very satisfied with both the app and testing experience. Adherence to testing significantly differed by age; however, the usability and acceptability of at-home testing and the MyDataHelps app did not differ by age, race/ethnicity, or gender. The majority of patients aged ≥65 years belonged to the high adherence group, whereas the proportion of participants with high adherence was lower among those aged 18-39 years and 40-64 years. The COVID-19 pandemic has hit those aged ≥65 years the hardest, with mortality rates over 60 times higher among those aged ≥65 years than those aged ≤54 years [18]. The difference in adherence by age group may reflect differences in risk perception influencing testing behaviors. Additionally, participants’ interpretation of the test results showed significant reliability with the study team’s interpretations, further demonstrating the feasibility of using self-tests outside the clinical environment. Participants were very capable of administering, reading, and reporting test results at home without clinical assistance.

### Comparison With Prior Work

Although many previous studies have analyzed the performance of rapid antigen tests for the detection of SARS-CoV-2, few studies have looked at users’ testing behavior. It is important to understand who uses rapid antigen tests, when people use rapid antigen tests, and how people test to facilitate the development of effective testing interventions. Nguyen et al [5] found that, among 31 employees of a large company, a daily serial rapid antigen testing intervention with an associated mobile app was highly acceptable, with mean adherence of 88% over a 21-day period. This finding is similar to our own findings of adherence, with over 60% of participants displaying high adherence (>75%) during the 14-day study period. Although the study by Nguyen et al [5] was nested in an employer testing program, with weekly COVID-19 testing required as a condition for employment, our study was based among households residing in 2 large metropolitan cities. The consistency of daily testing adherence across these 2 populations is notable and adds to the external validity of these results. Nguyen et al [5] also found that the acceptability of daily testing was related to the perceived threat of COVID-19, and participants were more likely to find daily testing acceptable in times of high SARS-CoV-2 prevalence. Our study was conducted prior to the widespread distribution of vaccines for SARS-CoV-2; therefore, it is possible that the perceived threat of COVID-19 was generally high throughout the population, contributing to high acceptability and adherence. It is important to reassess COVID-19 testing behaviors as the pandemic continues to evolve to understand the motivations and challenges with testing.

Additionally, the COVID-19 pandemic has escalated prepandemic health care disparities within the United States, and geographic inequities in COVID-19 incidence and testing availability persist [19-21]. Non-English speakers, persons of color, and those of lower socioeconomic status are less likely to have access to testing for SARS-CoV-2 than their counterparts, despite simultaneously having an increased proportion of positive cases and mortality [22,23]. Bringing health care services outside the traditional clinical environment offers solutions to accessibility, as well as bridging the gap to populations who have been systematically exploited by the health care system. In this study, we found that the acceptability and usability of at-home testing was consistent across all race/ethnicity categorizations, indicating that at-home testing could be a promising tool in addressing COVID-19 disparities.

As individuals navigate the return to work and school in the age of COVID-19, it is important that individuals have access to frequent and rapid testing to guide social engagement [24]. However, more information is needed on the diagnostic capabilities and limitations of these tools to ensure that individuals interpret the implications of their test results properly [25]. Additionally, although participants were asked to adhere to a 14-day continuous testing schedule for the purpose of this study, it is important to investigate further optimal testing schedules for SARS-CoV-2 detection [11]. We must also continue to evaluate the accessibility of at-home testing and the MyDataHelps app among diverse communities, including non-English speakers [26].

### Strengths and Limitations

This is the first study to look at the feasibility of at-home Ag-RDTs, filling an important gap in the literature. The strengths of this study include the longitudinal design, which allowed us to analyze adherence over time, and the use of a digital app for testing assistance and survey administration. The use of a digital app allowed participants to engage in the study from their homes, decreasing the burden of participation. Additionally, the wide inclusion criteria allowed the enrollment of a diverse cohort. However, this study is not without limitations. Study participants were required to speak English and have access to a smartphone, which limits the generalizability of our findings. Over 85% of adults in the United States own a smartphone; however, smartphone users vary from nonsmartphone users in terms of education, income, and age [27]. Additionally, only rapid antigen tests using nasal specimen collection were analyzed in this study; therefore, additional work may be necessary to evaluate the feasibility and acceptability of alternative SARS-CoV-2 testing modalities.

### Conclusions

As society establishes a new normal amid an ongoing pandemic, the development of accurate and rapid diagnostics is necessary to help curb SARS-CoV-2 disease transmission and safely navigate social re-engagement. The use of self-tests for COVID-19 with the MyDataHelps app for testing assistance was shown to be a feasible and accessible testing modality across gender, age, and racial groups, and more investigations into the efficacy of these testing modalities is indicated.

## Appendix

Multimedia Appendix 1 Screenshots of the MyDataHelps app.

## Abbreviations

Ag-RDTs: antigen-based rapid diagnostic tests
UMass: University of Massachusetts
