# Supplementary material for: Feasibility of At-Home Serial Testing Using Over-the-Counter SARS-CoV-2 Tests With a Digital Smartphone App for Assistance: Longitudinal Cohort Study
Source: JMIR Form Res. 2022 Oct 18;6(10):e35426. doi: 10.2196/35426 (PMC9580993; doi:10.2196/35426)
Supplement: Multimedia Appendix 1 [file formative_v6i10e35426_app1.pptx]

## Slide 1
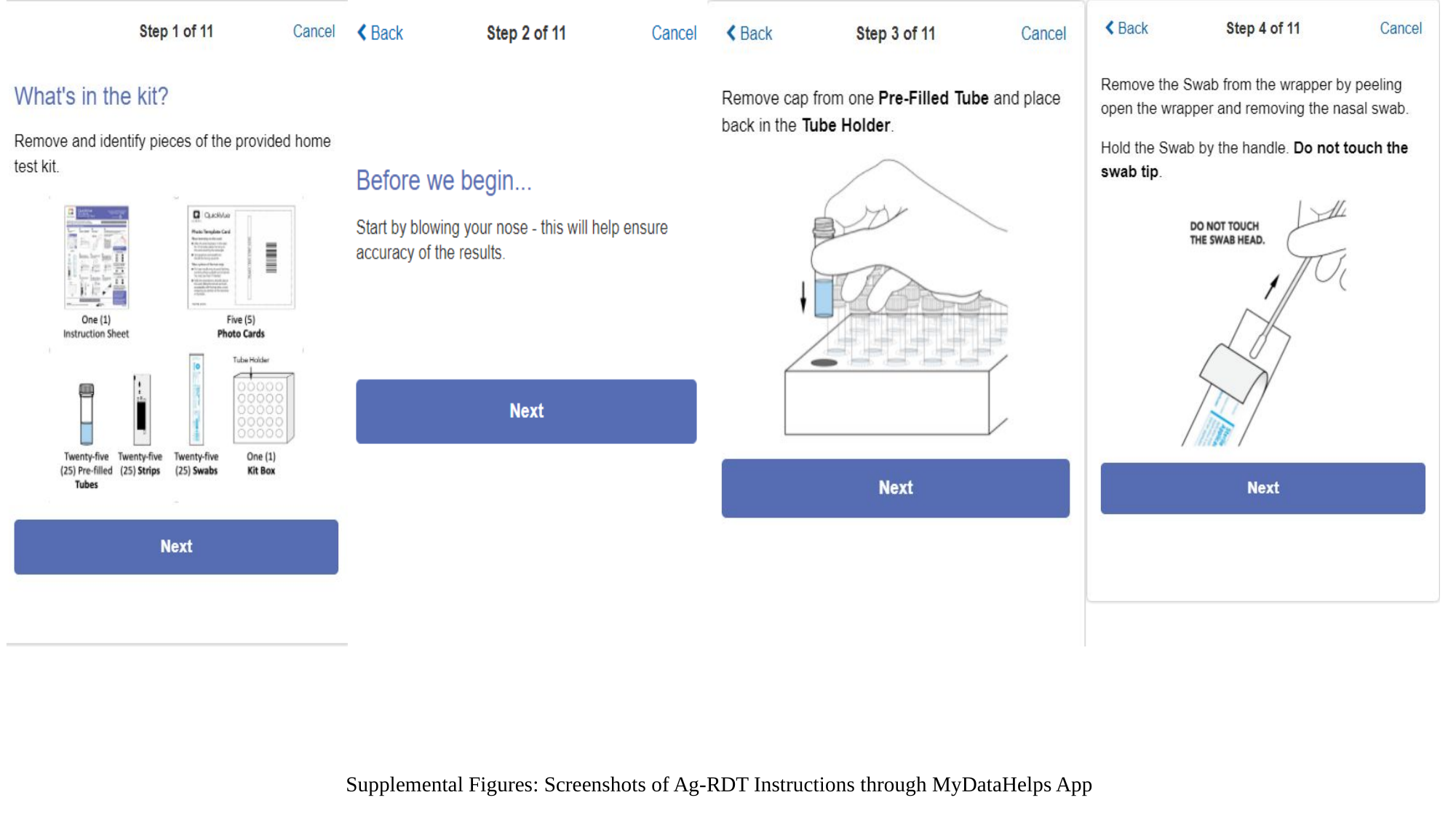

#
Supplemental Figures: Screenshots of Ag-RDT Instructions through MyDataHelps App

## Slide 2
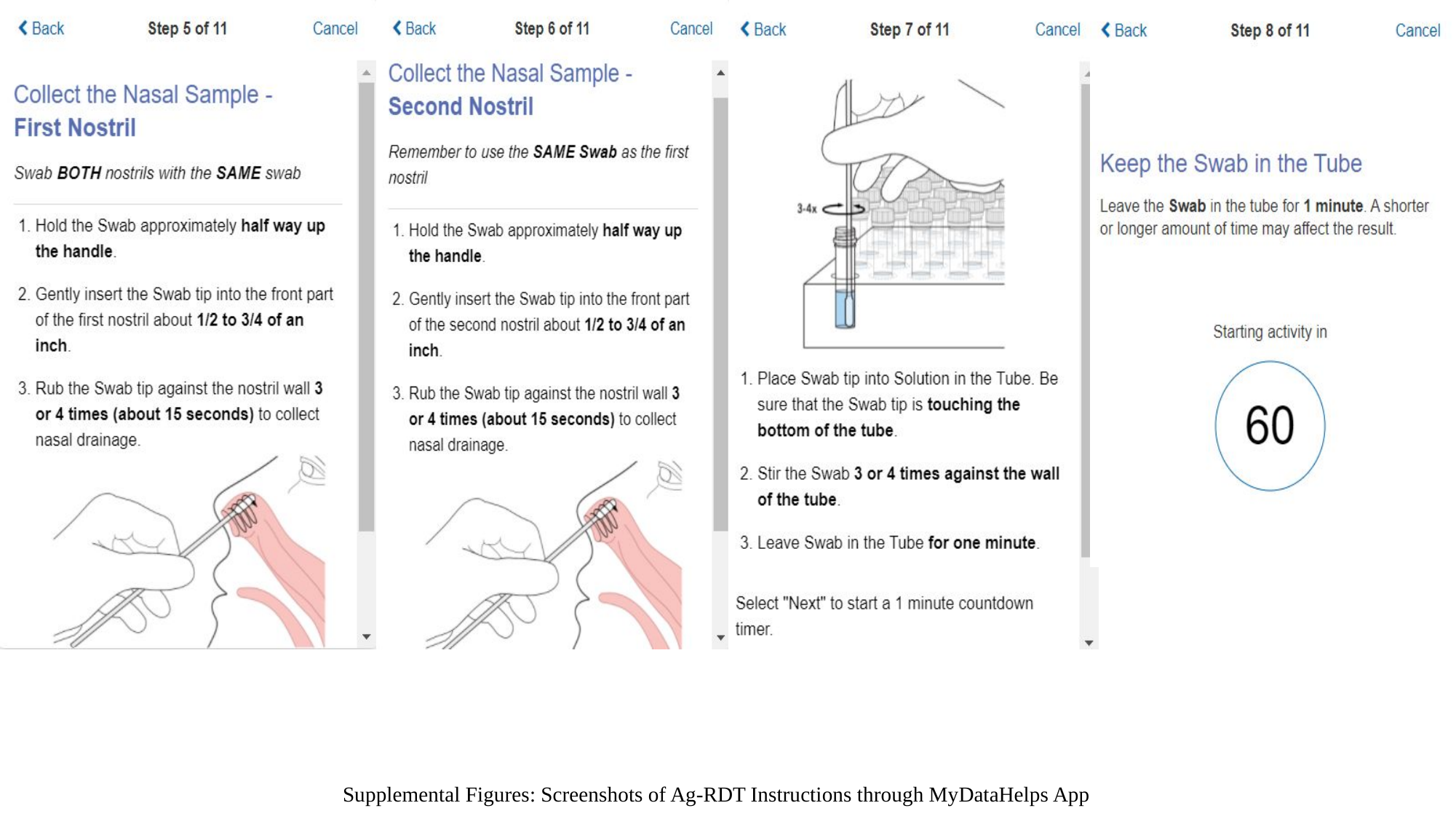

#
Supplemental Figures: Screenshots of Ag-RDT Instructions through MyDataHelps App

## Slide 3
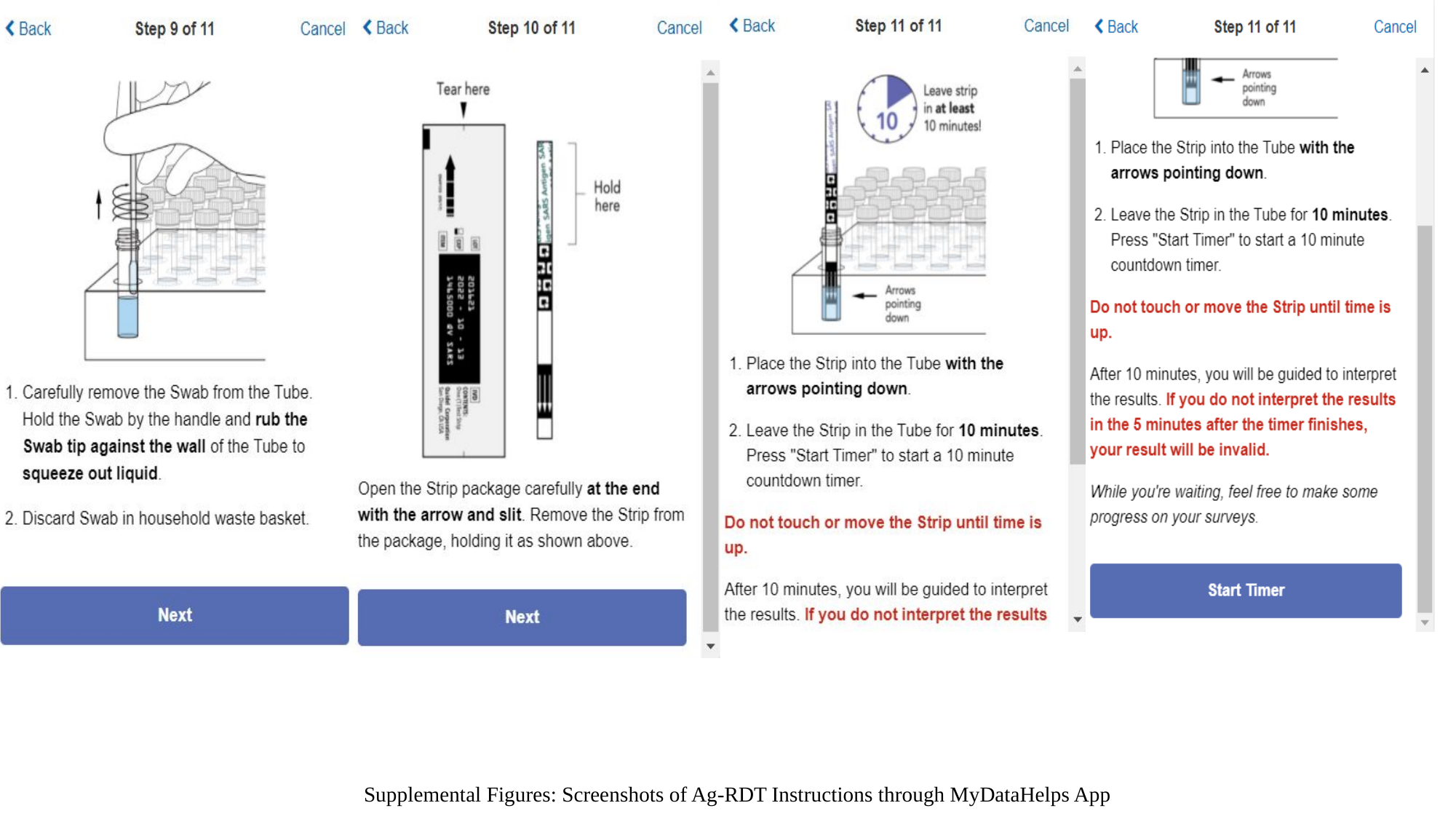

#
Supplemental Figures: Screenshots of Ag-RDT Instructions through MyDataHelps App

## Slide 4
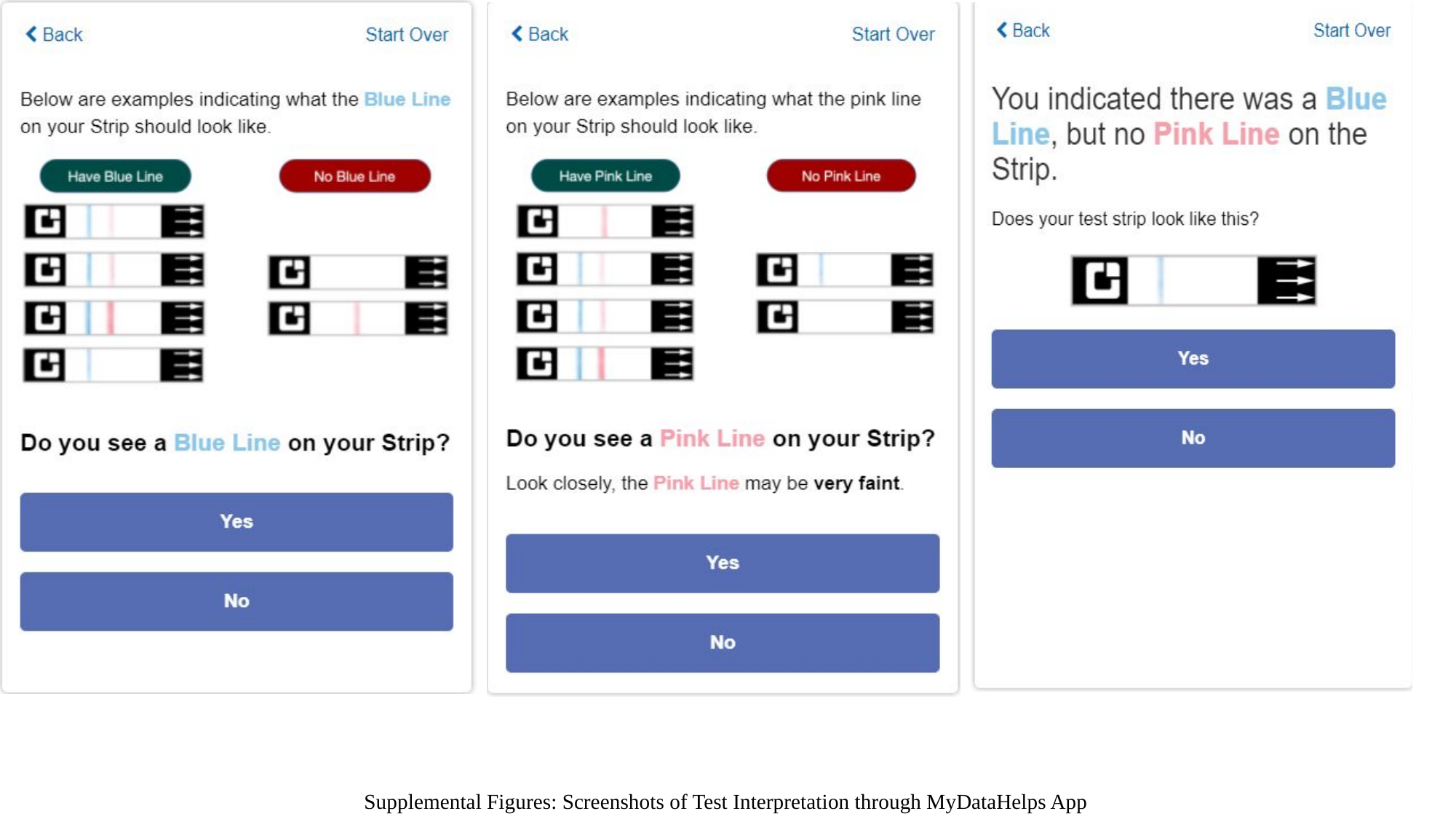

Supplemental Figures: Screenshots of Test Interpretation through MyDataHelps App
